# Spatial spread of the West Africa Ebola epidemic

**DOI:** 10.1098/rsos.160294

**Published:** 2016-08-03

**Authors:** Andrew M. Kramer, J. Tomlin Pulliam, Laura W. Alexander, Andrew W. Park, Pejman Rohani, John M. Drake

**Affiliations:** 1 Odum School of Ecology, College of Veterinary Medicine, University of Georgia, Athens, GA 30602, USA; 2 Department of Infectious Diseases, College of Veterinary Medicine, University of Georgia, Athens, GA 30602, USA

**Keywords:** disease ecology, Ebola, epidemiology, gravity model, network model

## Abstract

Controlling Ebola outbreaks and planning an effective response to future emerging diseases are enhanced by understanding the role of geography in transmission. Here we show how epidemic expansion may be predicted by evaluating the relative probability of alternative *epidemic paths.* We compared multiple candidate models to characterize the spatial network over which the 2013–2015 West Africa epidemic of Ebola virus spread and estimate the effects of geographical covariates on transmission during peak spread. The best model was a generalized gravity model where the probability of transmission between locations depended on distance, population density and international border closures between Guinea, Liberia and Sierra Leone and neighbouring countries. This model out-performed alternative models based on diffusive spread, the force of infection, mobility estimated from cell phone records and other hypothesized patterns of spread. These findings highlight the importance of integrated geography to epidemic expansion and may contribute to identifying both the most vulnerable unaffected areas and locations of maximum intervention value.

## 1. Introduction

The 2013–2015 epidemic of Ebola virus disease in West Africa is the largest documented, affecting more than 25 000 persons in three countries [1–3]. Large-scale epidemics inevitably affect heterogeneous populations, involving transmission between rural and urban communities, across national borders and between sub-populations that vary in contact structure, prior exposure and immunity, access to healthcare, and behaviour. Particularly, densely populated urban areas may function as persistent reservoirs by maintaining local chains of transmission [4,5], transportation hubs that connect scattered peripheral communities [6] and sources for long-distance movements [7]. In general, the problem of identifying key populations that disproportionately initiate, catalyse and sustain epidemic expansion is unresolved.

Data on the spatial spread of an emerging infection may be used to identify what spatial heterogeneities are important to epidemic expansion and assess the effectiveness of transport restrictions and other national policies [8–11]. Emerging pathogens have displayed a variety of spread patterns [12–15], which can determine not only the rate of spread, but also eventual geographical extent and suitable interventions. Thus, a better understanding of the spatial pattern of spread in the recent Ebola epidemic will provide knowledge useful for controlling future outbreaks and insight into how such analyses might be performed in real time.

**Figure 1.**
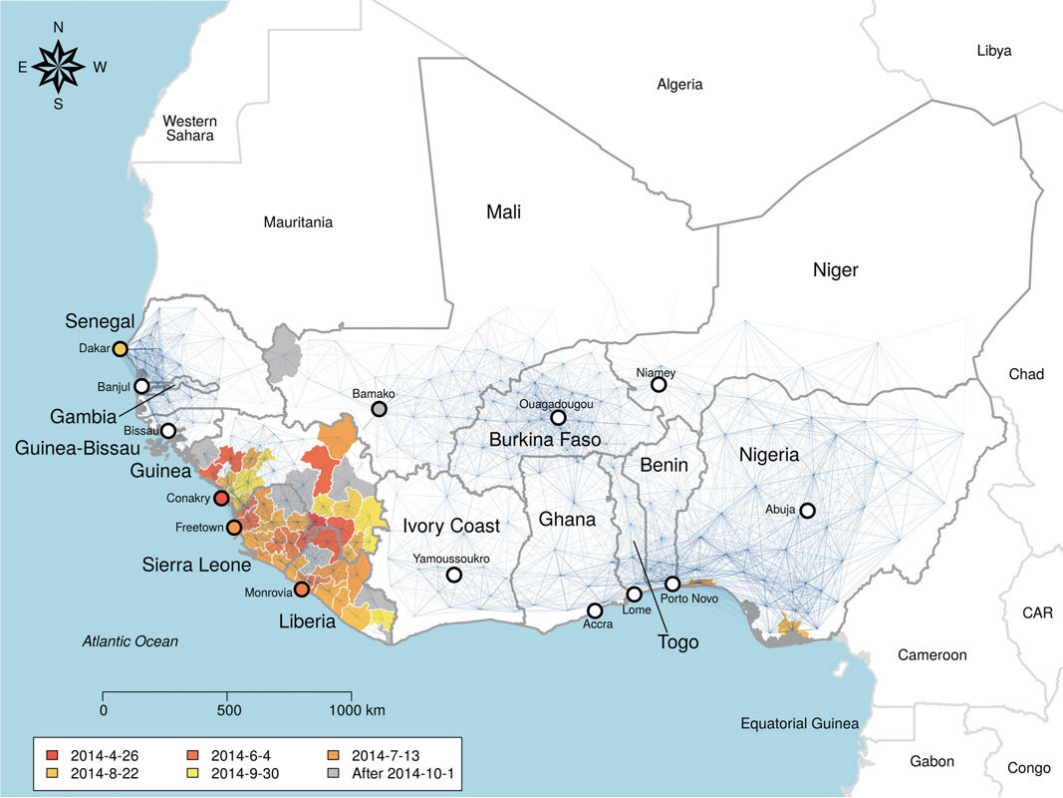
Network of Ebola virus transmission probability in West Africa. Blue lines represent the transmission probability for the best-fit model, which includes effects of distance, population density and whether links cross the border surrounding Guinea, Liberia and Sierra Leone. Thickness is proportional to transmission probability, nodes are administrative unit centroids. The map is fully connected and invisible links represent orders of magnitude lower transmission probability. Administrative units used to fit the model are coloured by date of infection. Infections recorded after 1 October are coloured grey and were not included in the model fit.

We characterized spread of Ebola in West Africa from April 2014 to September 2014 and estimated the time-varying outbreak hazard in locations across West Africa using network models. Such models have previously elucidated the spatial dynamics of human and animal pathogens [12,16,17]. Existing spatial analyses of the 2014 Ebola outbreak have either focused on single countries [8,18] or potential spread via air travel networks [9]. Other studies taking a network approach have focused on the individual scale contact networks underlying transmission [19,20]. Our model assumed a fully connected network where nodes represent geopolitical administrative units and weighted links represent the strength of potential infection pathways (figure 1). Sixteen competing hypotheses were represented by equations that differentially weight network links and incorporate the effects of factors such as distance between locations and population density [16]. Our analysis estimated the spatial transmission process from observed data available during the outbreak, while explicitly quantifying the risk to all locations on a daily time scale. We found that the probability of transmission between locations depended on distance, population density and international border closures between Guinea, Liberia and Sierra Leone and neighbouring countries, while available proxies for human mobility from individual cell phone records [11,21] were a poor predictor of spatial spread.

## 2. Material and methods

### 2.1. Model summary

Date of first infected case for administrative units across the core countries of Liberia, Guinea and Sierra Leone, and the surrounding nations of West Africa, was obtained from openly accessible sources during the outbreak and used to fit 16 spatial models reflecting effects of population density, border closure, long-distance dispersal and individual movement (see the electronic supplementary material, Methods) on daily transmission rates. Administrative units were taken to be the geographical scale of reporting in the core countries of Guinea (prefectures), Liberia (counties) and Sierra Leone (districts). Similar-sized units were used in the rest of the region (see Geographical data), collectively defining the nodes of the network. We fitted the models to the period of rapid spread, beginning with the second wave of the outbreak (26 April 2014) and ending on 1 October 2014 (electronic supplementary material, table S1). The data used for fitting were those available before or during December 2014. Model complexity varied from one free parameter (in null models lacking a spatial component) to six parameters. Akaike's information criterion (AIC) was used to quantify model performance.

### 2.2. Geographical data

Digital maps of the administrative unit boundaries of countries in West Africa were gathered from the Global Administrative Areas database (GADM) [22]. The administrative units used for analysis were determined by the scale of reporting in Liberia (county, GADM Level 1), Guinea (prefecture, GADM Level 2) and Sierra Leone (district, GADM Level 2). To provide relatively comparable administrative unit sizes across West Africa, Level 2 units were used in Mali and Senegal and Level 1 units in the other countries considered (Benin, Burkina Faso, Ivory Coast, Gambia, Ghana, Guinea-Bisseau, Niger, Nigeria and Togo; figure 1). The population size for each of these units was estimated from the most current WorldPop datasets for each country [23] (www.worldpop.org.uk/ebola/) by aggregating estimated population sizes using the raster package v. 2.2–31 [24] in R. v. 3.0.3 [25]. Distances between units were calculated from the polygon centroids.

### 2.3. Mobility data

Human mobility data have been estimated for West Africa by the Flowminder project [26]. The Flowminder dataset provided estimates of mobility between locations using models fitted to data from two sources, national census microdata on annual changes in residence and mobile phone call detail records [21]. These within-country movements were extrapolated to unmeasured countries and between-country movements using gravity models similar to the one we fitted here [21]. The relatively poor spatial and temporal resolution means these models represent significant extrapolations rather than direct measurement [21]. Nevertheless, these were the best existing estimates for movement at the relevant scale for this region. The downloaded data were merged with the list of administrative units created above to create a unified dataset. These data (archived with the code on the Dryad Data Repository: http://dx.doi.org/10.5061/dryad.n3g63) are stored as a matrix of the estimated flows between each pair of locations. Ideally, these estimates are a predictor of the magnitude of linkage between each pair of locations. This matrix was used in model fitting in place of, or in addition to, the matrix of distances (see Transmission models). The analyses here used the census microdata model, Senegal mobile phone model and Ivory Coast mobile phone model [21].

### 2.4. Disease reports

Dates of first confirmed infections for each administrative unit were compiled from WHO reports and country-level reports (electronic supplementary material, table S1). Infection dates were classified as part of either the first wave (prior to May) or second wave of the epidemic. Two locations in Guinea are thought to have maintained infection from the first wave to the second: Guéckédou and Conakry. For this analysis, these were treated as the initially infected locations on 26 April 2014, the date on which a new case appeared in Conakry following a period of no new infections at the end of the first wave (electronic supplementary material, table S1). Health officials in Guinea later indicated that the second wave was linked to infection in Conakry [27]. We also analysed the data starting with the initial date of infection in Guéckédou (12 December 2013), but results were qualitatively similar, and analysing spread and recovery in the first wave, as well as subsequent reinfection in this second wave, is beyond the scope of this paper. Administrative units with confirmed infections prior to 1 October 2014 are included, with infection status and confirmed first infection reflecting revisions up to that date. In Liberia, Guinea and Sierra Leone there were 43 infected units out of 63, and 46 infected out of 290 in West Africa. The latest infection date, and hence the end date for model fitting is 30 September 2014 (Lola prefecture, Guinea). The data were compiled and adjusted during the outbreak, with the final data representing public knowledge in December 2014.

### 2.5. Transmission models

Models adopted a compartmental structure similar to epidemiological Susceptible-Infected models [16]. Administrative units were assumed not to have recovered in the time period considered (26 April 2014 to 30 September 2014) and hence remained potential sources of new infections. This assumption fitted the rapid expansion of the disease over this period and the fact that disease-free conditions were not declared in any locations until after this date, many of which subsequently reported additional infections—although not in Senegal and Nigeria (due to the rapid isolation and better public health infrastructure in these countries). Dynamics within each unit and their effect on spread to other counties were modelled implicitly, allowing us to characterize the broad geographical features driving spread without making unnecessary assumptions concerning the mechanisms of local transmission. Examination of corrected case reports following the outbreak indicates that the number of confirmed and suspected cases was strongly related to district population size (electronic supplementary material, figure S1).

Two highly simplified models of non-geographical spread were fitted as null hypotheses. The simplest model (1) fitted a constant risk of infection for all administrative units (electronic supplementary material, tables S2 and S3), while the probability of infection in the (2) well-mixed model depended on the number of counties infected independently of their location and population size (electronic supplementary material, tables S2 and S3). A set of increasingly complex spatial models were fitted with the probability of infection between all susceptible units *i* and sources *j* as a logistic function of the Euclidean distance between centroids *d_*ij*_*, the population distribution of the units *p*, the presence of a border, individual mobility data, or some combination of these (electronic supplementary material, tables S2 and S3). Two models depended only on geographical distance: (3) diffusive spread with probability of infection decaying exponentially with distance *d_*ij*_* from infected units (electronic supplementary material, tables S2 and S3),

2.1 $$\frac{1}{1+e^{\beta_{0}+\beta_{1}d_{ij}}},$$

and (4) diffusive spread with allowances for long-distance dispersal via a fitted parameter as an exponent of the distance between units (electronic supplementary material, tables S2 and S3).

2.2 $$\frac{1}{1+e^{\beta_{0}+\beta_{1}{d_{ij}}^{\beta_{2}}}}.$$

To test the hypothesis that population density altered risk of infection we fitted four models that included distance and population density: (5) a model with a gravity term [16] where the connection between units *i* and *j* was weighted by the population size *p* in each (electronic supplementary material, tables S2 and S3)

2.3 $$\frac{1}{1+e^{\beta_{0}+\beta_{1}(d_{ij}/{\left(p_{i}p_{j}\right)}^{B_{2}})}},$$

(6) the previous gravity model with allowances for long-distance dispersal (see (3), electronic supplementary material, tables S2 and S3),

2.4 $$\frac{1}{1+e^{\beta_{0}+\beta_{1}({d_{ij}}^{\beta_{2}}/{\left(p_{i}p_{j}\right)}^{B_{3}})}},$$

(7) a force of infection model from [16] (modified from [28], electronic supplementary material, tables S2 and S3),

2.5 $$1-e^{\left(\beta_{0}{p_{i}}^{\beta_{1}}\sum_{j}({p_{j}}^{B_{2}}/{d_{ij}}^{\beta_{3}})\right)\left(\mathrm{no}\;\mathrm{network}\right)},$$

(8) a normalized force of infection model ([16], electronic supplementary material, tables S2 and S3),

2.6 $$1-e^{\left(\beta_{0}{p_{i}}^{\beta_{1}}\sum_{j}({p_{j}}^{B_{2}}/{d_{ij}}^{\beta_{3}})/{\left(\sum_{k\neq i}({p_{k}}^{B_{2}}/{d_{ik}}^{\beta_{3}})\right)}^{\beta_{4}}\right)\left(\mathrm{no}\;\mathrm{network}\right)},$$

(9) a radiation model that takes into account the population distribution surrounding the source location using the radiation function rad from [16,29] (electronic supplementary material, tables S2 and S3),

2.7 $$\frac{1}{1+e^{\beta_{0}+\beta_{1}d_{ij}+\beta_{2}\mathrm{rad}}},$$

and (10) a population-weighted opportunities model that takes into account the population distribution surrounding the destination location using function pwo as in reference [30] (electronic supplementary material, tables S2 and S3).

2.8 $$\frac{1}{1+e^{\beta_{0}+\beta_{1}d_{ij}+\beta_{2}\mathrm{pwo}}}.$$

These models represent various hypotheses about how distance and density might drive spatial transmission, and therefore are implicitly fitting mobility among units. As an alternative, we proposed a set of models based on previously estimated mobility patterns (estimated with a gravity model [21] similar to Model 5): (11) transmission probability depends on models (mob) fitted to Senegal call data records (electronic supplementary material, tables S2 and S3)

2.9 $$\frac{1}{1+e^{\beta_{0}+\beta_{1}\mathrm{mob}}},$$

and (12) transmission depends on distance and the modelled mobility (electronic supplementary material, tables S2 and S3).

2.10 $$\frac{1}{1+e^{\beta_{0}+\beta_{1}\mathrm{mob}+\beta_{2}d_{ij}}}.$$

The models fitted to Senegal call data records were the best performing out of models fitted to Ivory Coast call data records, Senegal call data records and census microdata [26].

The models were fitted to the 63 administrative units in Liberia, Guinea and Sierra Leone and to all the administrative units in West Africa. Since transmission rates and patterns may differ between countries due to differences in transport infrastructure, cultural practices and healthcare interventions, including border closure [31], we fitted four additional models to test for these hypothesized effects (see Results and electronic supplementary material, tables S2 and S3). In the gravity and diffusion models, we tested whether transmission was reduced across international borders among any two countries or whether transmission from the core of Liberia, Guinea and Sierra Leone to other countries was reduced (electronic supplementary material, tables S2 and S3). The first of these options assumes all borders may have acted as a deterrent to the spread of Ebola, while including only the effect between the core and non-core countries better represents that actual isolation of the infected countries by the rest of the region (e.g. [32]). We were not able to include the actual date of border closure, but there were widely documented restrictions on passage out of the core countries ranging between March and August 2014.

### 2.6. Model fitting

The models described above were fitted as discrete time models with a daily time step to the data on first infection dates using maximum likelihood (ML). These models inherently imply that the graph of transmission is fully connected and symmetric (except for the radiation and population-weighted opportunity models, which are asymmetric), although the flow of transmission among locations is highly heterogeneous. ML parameters were obtained using a combination of downhill simplex (Nelder–Mead) and simulated annealing. Parameters were sought that best reconciled the daily observations of administrative units either remaining uninfected or becoming infected on a given day (and thereby joining the pool of administrative units acting as infectious sources for future days). On each day, *t*, the set of source units, *j*, were identified, and uninfected nodes *i* grouped into two subsets: ‘*i*_1_: infected at time *t*’ and ‘*i*_0_: remaining uninfected at time *t*’. Consequently, the likelihood of observing events on day *t* is the product of the probabilities of all *i*_0_ escaping infection from all *j* multiplied by the probabilities that all *i*_1_ are infected by at least one *j*. We let *P_*ij*_* be the probability that node *j* infects node *i* (at implicit time *t*), with the form of *P_*ij*_* given by the transmission models in electronic supplementary material, tables S2 and S3. Since the set of uninfected nodes, *i*, at time *t* is conditional on having remained uninfected until *t*, *P_*ij*_* is the discrete time hazard of infection [33]. The model is therefore a type of survival model, where the hazard function for any given administrative unit is both time varying and a function of the states of the other units in the population. To accommodate these properties, the data were coded as daily records of infection status (infected: *y* = 1; uninfected: *y* = 0) and fitted to minimize the negative log-likelihood:

2.11 $$-\sum_{t}\log\left\{\prod_{i_{1}}\left[1-\prod_{j}\left(1-P_{i_{1}j}\right)\right]\times \prod_{i_{0}}\left[\prod_{j}\left(1-P_{i_{0}j}\right)\right]\right\}.$$

In this expression, the outermost sum is over all dates in the portion of the time series used for fitting. The expression in curly braces consists of two factors corresponding to the possible outcomes for sites remaining uninfected up to *t*. The first of these is the likelihood of infection for all sites infected on *t*, obtained by enumerating the probability of escaping infection from all sources and taking the complement. The factor corresponds to the likelihood of escaping infection for all sites remaining uninfected.

Wald-type confidence intervals for the ML parameters were estimated by solving for (*q ×* sqrt(diag(*A*)))/*k* as in [34] where *q* is the 95th equicoordinate quantile obtained with the R function qmvnorm (package: mvtnorm [35]), *A* is the variance–covariance matrix of the likelihood surface estimated by inverting the Hessian (package: numDeriv [36]) at the ML point, and *k* is the number of parameters in the model. However, we note that for some models the Hessian was not invertible (electronic supplementary material, table S4).

### 2.7. Simulation and validation

To quantify goodness of fit, we compared simulated spread to the observed data. We simulated stochastic spread from Guéckédou and Conakry for 1 year from the beginning of the outbreak. From 1000 simulations of each model, we assessed the median and prediction intervals for unit infection rate, maximum and median spread distance from Conakry, and the area of infection. The simulations also provided a median date of infection and 95% prediction interval for each county in the network that was compared to the observed infection time series and to out-of-fit predictions for the units infected after 1 October 2014.

## 3. Results

The observed spread of Ebola virus was best fitted by a gravity model with decreased transmission from the core countries to the rest of the region with link weights

3.1 $$P_{ij}=\left\{\begin{array}{cc} \beta_{3}\frac{1}{1+e^{\beta_{0}+\beta_{1}(d_{ij}/{\left(p_{i}p_{j}\right)}^{\beta_{2}})}} & \mathrm{if}\;\mathrm{crossing}\;\mathrm{core}\;\mathrm{border}\\ \frac{1}{1+e^{\beta_{0}+\beta_{1}(d_{ij}/{\left(p_{i}p_{j}\right)}^{\beta_{2}})}} & \mathrm{if}\;\mathrm{not}\;\mathrm{crossing}\;\mathrm{core}\;\mathrm{border} \end{array}\right\},$$

where *β*_0_ determined the background infection rate, the distance between two units (*d_*ij*_*) was multiplied by a coefficient (*β*_1_), and the gravity term was the product of the population sizes (*p_*i*_p_*j*_*) modulated by an exponent (*β*_2_) (electronic supplementary material, table S2). A conditional multiplier (*β*_3_) set the effect of crossing a border of the core countries. In this model, probability of transmission declined with distance between nodes, increased with the population density of the nodes and was lower when transmission occurred between any of the three core countries and the remaining countries in West Africa (figure 1; electronic supplementary material, table S3). The resulting transmission network had the highest transmission risk within countries and across borders of contiguous countries and very weak long-distance links (figure 1). Low risk of transmission from the core countries to the rest of the network was due not only to the border penalty (*β*_3_), reducing transmission by approximately sixfold, but also to low population density in the border regions. Among competing models, one that treated any border, including the borders between the three core countries, as a potential barrier to transmission was ranked within a cluster of models with less support (ΔAIC > 2; electronic supplementary material, table S2), suggesting that the measurable effects of borders were only between the core and non-core countries.

Model comparison suggests that human population density was a critical factor in spatial spread; the second and third-ranked models were the gravity model with no effect of borders and a gravity model with a power term allowing for increased long-distance dispersal (electronic supplementary material, table S2). The small range of very high correlations (*r* = 0.96–0.99) in link weights implies that the three models made similar predictions about relative and absolute transmission probabilities despite differences in mechanistic motivation (electronic supplementary material, table S5). Importantly, these models agreed that transmission rate increases with population density at both source and destination nodes. Models with less support, including the diffusion and radiation [29] models, were less strongly (but still significantly) correlated with the best-fit models (*ρ *= 0.81–0.91). By contrast, some mechanistically plausible models provided unequivocally poor fits to the observed pattern of spread (electronic supplementary material, table S2, figures S2 and S3). Models assuming constant and equal infection probability from any infected node were inferior (electronic supplementary material, table S2). We also found significantly lower support for more complicated models of mobility that have elsewhere been proposed as alternatives to gravity models [28,30]. Perhaps most surprisingly, we found that models based on individual movement estimated from cell phone records in Senegal and Ivory Coast [11,26], and census microdata records [21,26] (see Material and methods) performed poorly compared with gravity, diffusion and force of infection models (electronic supplementary material, table S2, figures S2 and S3).

**Figure 2.**
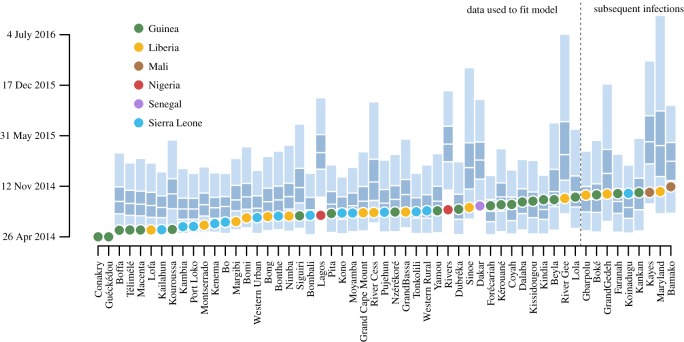
Predicted day of infection for infected administrative units based on the best-fit model. The predicted day of infection is based on simulations from 24 April. For each node, the 95% prediction interval is in light blue with the interquartile range specified as dark blue and the median infection as a break. Dots represent the observed day of infection coloured by country. Infection locations used in fitting are left of the dotted line and infections subsequent to 1 October are to the right of the dotted line. Observations were within confidence bounds except for the two Nigerian states and the prefecture of Kouroussa in Guinea, which were predicted to become infected later than observed.

Because the 16 models considered do not represent all possible models, it is important to confirm that the favoured models do a good job at recreating important features of the epidemic. The best fitting model predicted a time series of total administrative units infected that very closely matched observations (electronic supplementary material, figure S4). Additionally, two measures of infected area (median distance from Guéckédou, the presumed location of the index case, and the area of the convex hull of infected locations) also matched observations, while the maximum distance from Guéckédou increased faster than predicted by the model, implying that the model fails to capture some components of long-distance movement. At the smaller scale of individual administrative units, all except three (Kouroussa, Guinea; Lagos and Rivers, Nigeria) fall within the 95% prediction intervals of the best fitting model (figure 2; electronic supplementary material, figure S5). Particularly, spread to Dakar, Senegal was accurately predicted by the model. These prediction matches included predictions on units first infected after 1 October; these data were not used to fit the model. The best fitting model had the highest overlap between observations and prediction intervals and also had the highest correlation between observed date of infection and median simulated date of infection (electronic supplementary material, figure S3). The prediction intervals were narrower for the best-fit model, but still wide, emphasizing the importance that sequencing plays in time of infection. Early stochastic events alter the possible remaining paths, resulting in a wide variety of possible patterns when simulating from the starting conditions.

**Figure 3.**
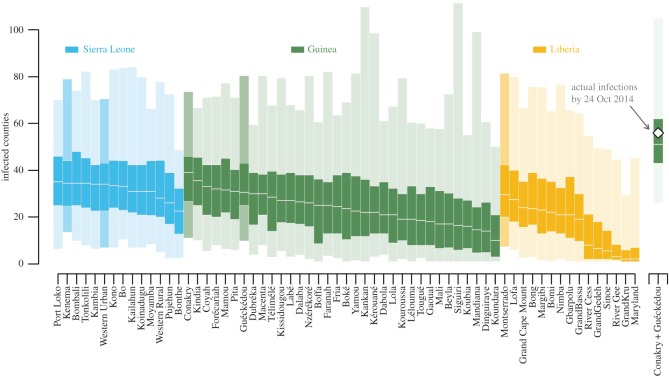
Dependence of spread on initial administrative unit infected. Shown is the predicted number of nodes infected at 6 months given infection starting in each of the units in the core countries. Each node is coloured by country and ordered by median epidemic severity, with the 95% prediction interval (1000 simulations) lightly shaded, the interquartile range darkly shaded and the median number of counties infected in white. On the far right are simulations from the observed scenario of joint spread from Guéckédou and Conakry and the observed number of infected units at 6 months. Important locations are darkened: Guéckédou and Conakry are population centres in Guinea and the initially infected areas in the second wave of the Ebola outbreak, Kenema was the location of an important treatment centre early in the outbreak, Freetown (Western Urban) and Monrovia (Montserrado) are the capitals and largest cities of Sierra Leone and Liberia, respectively.

Calibrated models then allowed us to predict what could have happened if the epidemic originated in another part of West Africa. Simulations of new epidemics with initial infections located in each unit in the core countries (figure 3) revealed how the realized epidemic path would have affected epidemic severity. Different starting points resulted in order-of-magnitude differences in median number of counties infected; however, 95% prediction intervals were much more similar, underscoring the large effect of intrinsic noise in this system. Decomposition by analysis of variance showed that starting location explains only 14% of the variation in epidemic size, i.e. the number of nodes infected over the time period. Moreover, simulating concurrent spread from two locations (Conakry and Guéckédou) was fully consistent with the observed speed of spread, suggesting that spread along two paths resulted in a substantially more widespread outbreak (figure 3).

## 4. Discussion

Our study found the spread of Ebola virus to be strongly affected by geographical features. Particularly, the probability of transmission between locations depended on distance, population density and international border closures between Guinea, Liberia and Sierra Leone and neighbouring countries. Other studies at more local scales [10,18] support the suitability of generalized gravity models to match observed patterns in the Ebola outbreak, but were not constructed to provide the contrasts studied here. Thus, we additionally found lower support for a variety of other hypothesized patterns of spread, including some proxies for human mobility estimated from individual cell phone records [11,21] which here were a poor predictor of spatial spread. Our fitted model was validated using hindcast simulations at both broad spatial scales and for the infection dates of specific localities. Further, we challenged the fitted model to make out-of-fit predictions (i.e. units first infected after 1 October 2014). The resulting model enabled us to project potential paths of transmission risk across West Africa and illustrated how the location of origin can greatly affect the speed of expansion and containability of an epidemic [37].

While all models assumed fit parameters to be constant for the time period considered, the best model nevertheless generated small-scale predictions for realized disease spread after 1 October that align well with data on administrative units infected after this time, showing the usefulness of this model even after the epidemic had peaked [38]. Because this model does not explicitly include information on the number of cases in administrative units [18] or other factors like treatment facilities [39], any discernible effects of these factors are instead included in the estimated effects of distance and population density. This may be a potential advantage of aggregated models for predicting spread and allocating resources, given probable differences in under-reporting over time and among countries. Yang *et al.* [18] used case reports and a model that accounts for under-reporting to find support for a similar gravity-type model of spread, and to further estimate the most likely spatial source of infection within Sierra Leone. However, in several cases their model suggests infections from outside the neighbouring districts [18], indicating the need to complement detailed local models with coarse-grained models such as described here. Further insight may be possible by combining these spatial models of transmission with genetic phylogenies that reveal additional details of the spatial pattern of spread [19,40–42], as has been done for multiple outbreaks in central Africa [43]. Because our model enables risk of spread to update in response to the propagating epidemic, it may offer a useful framework for updating risk in response to changes in human behaviour or interventions [20].

There are several explanations for the relatively low performance of models based on estimated mobility data from the Flowminder project [26]. These models have been extrapolated from nearby countries (Ivory Coast and Senegal) and as a result may not adequately represent movement patterns in the more rural core countries affected in the outbreak. Furthermore, movements across international borders are not directly measured in the cell phone data records, so these movements are also extrapolations [26]. Therefore, it is possible that estimates based directly on cell phone data records in Guinea, Liberia and Sierra Leone and between countries would have approximated the gravity model fit here, providing similar information. An alternative explanation is that movement patterns of patients suffering from Ebola virus disease are substantially different from everyday movement patterns, in which case the gravity models based on disease spread are capturing this difference. Finally, there may be some bias in the movement data that results from socio-economic differences between infected patients and the sample of cell phone data records, although we consider this less likely. Yang *et al.* [18] had similar success with the approach we used here, directly using distance and population size instead of trying to estimate interlocale commuter flows.

Importation risk at a larger (global) scale was previously estimated by combining air travel data with case reports [44] and by simulating coupled air travel, land travel and epidemic dynamics using an epidemic and mobility model [9]. Because the actual spatial spread was not used to develop either of these models they provide limited insight into any characteristics of spread specific to this outbreak and to the role of overland human movement during Ebola outbreaks. Importantly, two of three infected locations not predicted by our model were in Nigeria, the only air travel-assisted transmission known to have occurred in West Africa. Accordingly, our network models may have underestimated the risk due to air travel, indicating potential added value of air travel-based analyses [9]. Regression models fitted to data within the core countries of Guinea, Liberia and Sierra Leone [10] show that the number of cases, distance and population density are all correlated with transmission between locations, in agreement with our best-fit mechanistic model. However, our findings suggest their conclusion that borders are unimportant [10] may be incomplete because they do not distinguish between the reportedly porous borders within the core countries and between the core countries and neighbouring nations. Our inference of spread pattern within the core countries and to neighbours is robust, but further spread in the region is difficult to infer given the vastly different public health systems in surrounding countries. This is borne out in the quick isolation and control achieved in countries other than Guinea, Liberia and Sierra Leone. Because the bulk of local spread in our network is inferred from the core countries, predicted spread within non-core countries can be viewed as expectations in the absence of rapid isolation. Future work focused on the role of border closure [31] promises to integrate geography and policy aimed at mitigating spread, while minimizing negative effects on transport of aid and economic activity [45].

In conclusion, the models developed here provide novel insights into the pattern of spatial spread in the 2013–2015 West Africa Ebola outbreak. The analyses highlight possible weaknesses in the use of some available individual-based mobility data for forecasting Ebola virus spread. More generally, our model estimated pair-wise transmission risk, facilitating the identification of influential ‘hubs’ such as Conakry (figures 1 and 3). The results also suggest that stochasticity in the epidemic path would complicate prediction of actual dates of infection from the initial starting point even with a transmission model based on number of cases and reliable case reports. Because model fitting here is independent of the epidemic curve, requiring only the date of first infection, real-time updating and spatial forecasting are possible with minimal input data, providing a framework for rapid analysis of outbreaks. The combination of reduced transmission across borders between core and non-core countries and low population density along several of these borders (for instance, between Liberia and Ivory Coast) seems to have greatly reduced risk to neighbouring countries (figure 1). Ultimately, it was these fortuitous geographical features, combined with rapid isolation and control when cases were imported to Senegal and Nigeria, that prevented spread across the highly connected networks into more densely populated areas of West Africa.

## Supplementary Material

Electronic Supplementary Material: Figures and Tables

## Data accessibility

The datasets used in this study are available at Dryad under accession number http://dx.doi.org/10.5061/dryad.n3g63; the associated code for fitting and simulating models is also available there.
