# Supplementary material for: Spatial spread of the West Africa Ebola epidemic
Source: R Soc Open Sci. 2016 Aug 3;3(8):160294. doi: 10.1098/rsos.160294 (PMC5108957; doi:10.1098/rsos.160294)
Supplement: Electronic Supplementary Material: Figures and Tables [file rsos160294supp1.pdf]

Figure S1:
Number of confirmed and suspected cases of Ebola up to 30 September 2014 in districts of Guinea (black), Liberia (red) and Sierra Leone (green), as a function of district population size.

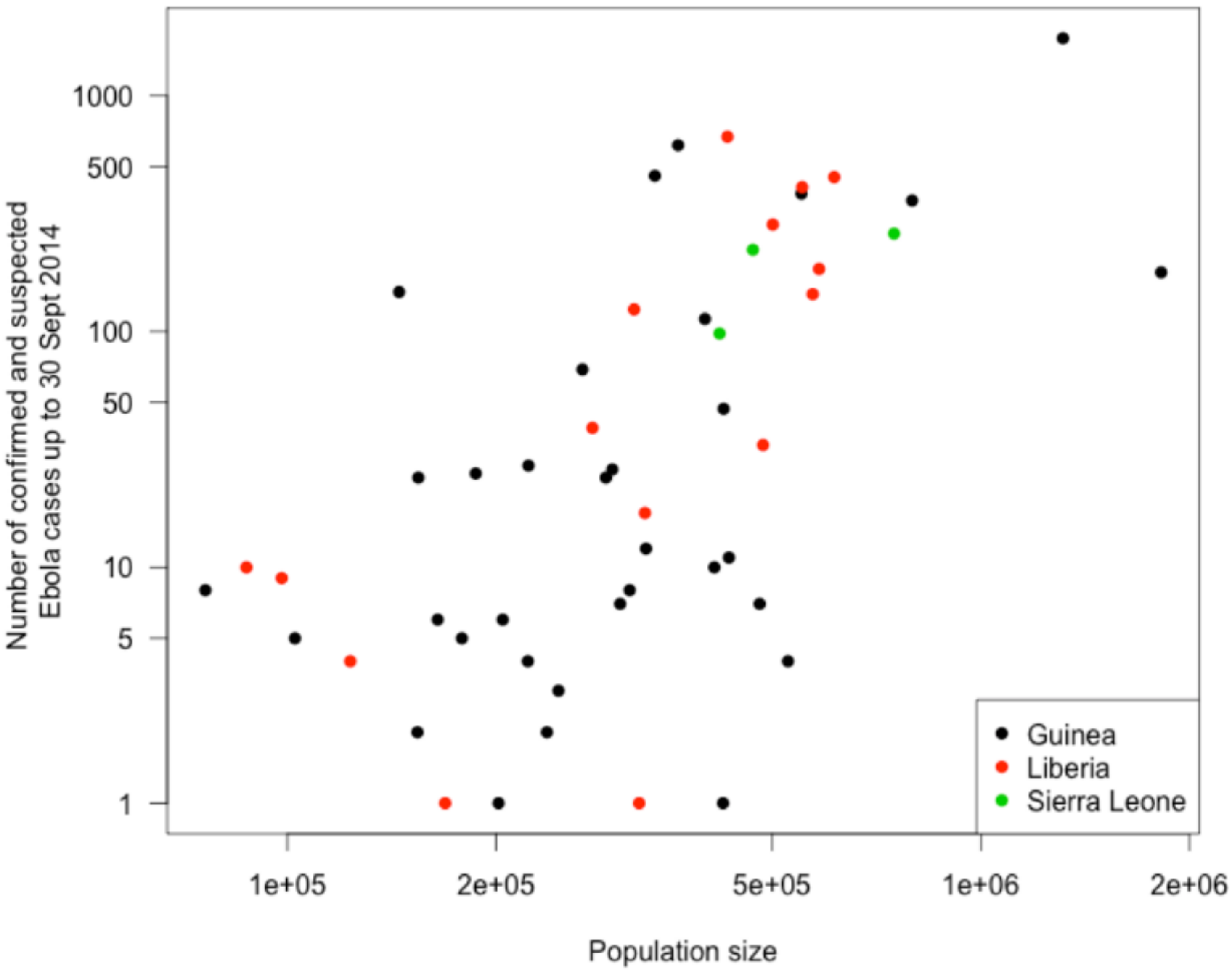

Number of confirmed and suspected
Ebola cases up to 30 Sept 2014
1000
500
100
50
10
5
1
1e+05
2e+05
5e+05
1e+06
2e+06
Population size
Guinea
Liberia
Sierra Leone

Figure S2:
Micro-scale fit of competing models. Predicted day of infection for infected administrative units based on each of the models (excluding best fit model, shown in Fig 2). The predicted day of infection for nodes based on simulations from April 24th. For each node, the 95% prediction interval is in light gray with the interquartile range specified as dark gray and the median infection as a break. Dots represent the observed day of infection for each node. Infection locations used in fitting are to the left of the dotted line and infection subsequent to October 1st are to the right of the dotted line.

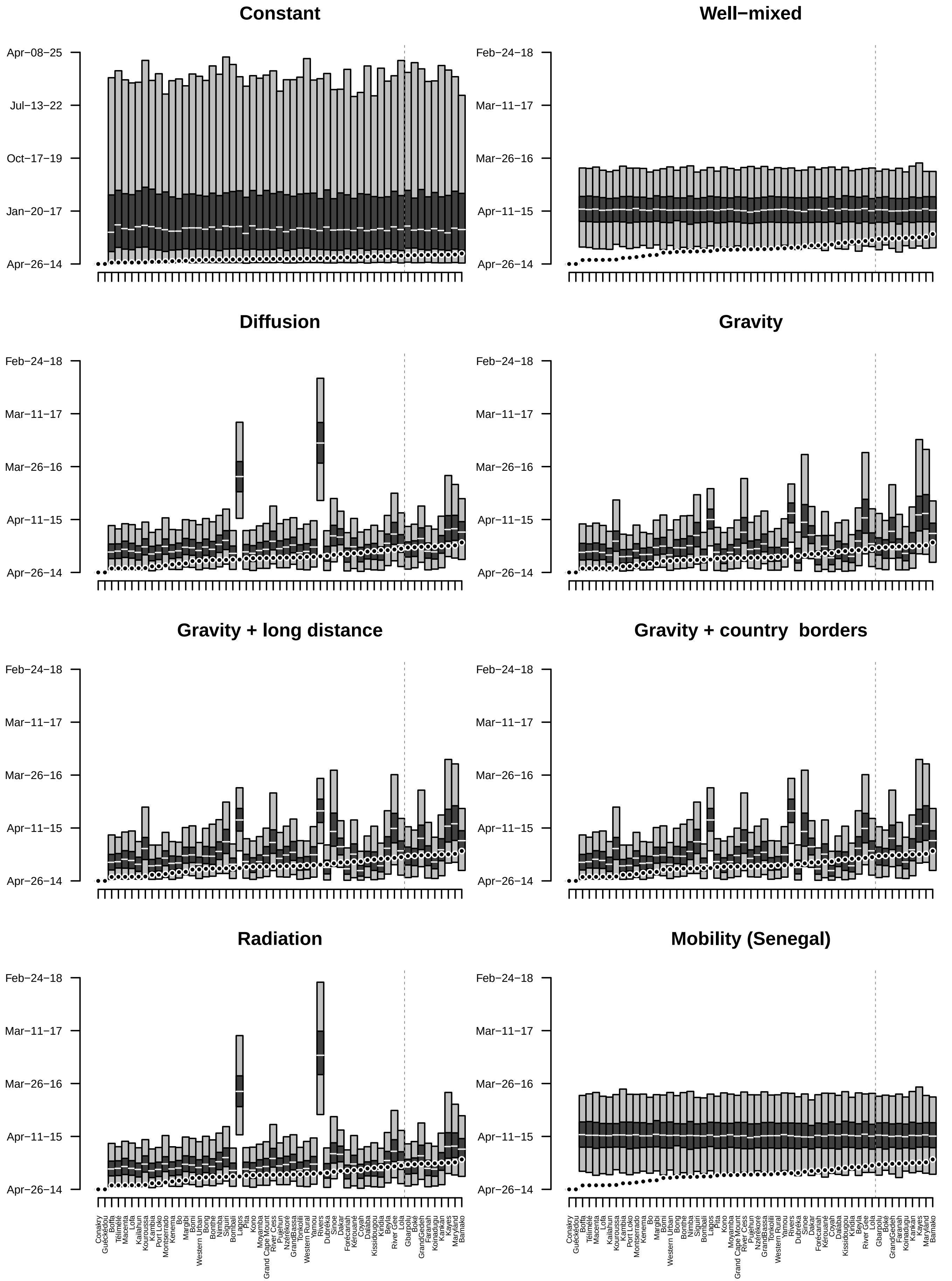
Constant
Well−mixed
Diffusion
Gravity
Gravity + long distance
Gravity + country borders
Radiation
Mobility (Senegal)
Apr−08−25
Jul−13−22
Oct−17−19
Jan−20−17
Apr−26−14
Feb−24−18
Mar−11−17
Mar−26−16
Apr−11−15
Conakry
Guéckédou
Boffa
Télimélé
Macenta
Lofa
Kailahun
Kouroussa
Kambia
Port Loko
Montserrado
Kenema
Bo
Margibi
Bomi
Western Urban
Bong
Bonthe
Nimba
Siguiri
Bombali
Lagos
Pita
Kono
Moyamba
Grand Cape Mount
River Cess
Pujehun
Nzérékoré
GrandBassa
Tonkolili
Western Rural
Yamou
Rivers
Dubréka
Sinoe
Dakar
Forécariah
Kérouané
Coyah
Dalaba
Kissidougou
Kindia
Beyla
River Gee
Lola
Gbarpolu
Boké
GrandGedeh
Faranah
Koinadugu
Kankan
Kayes
Maryland
Bamako

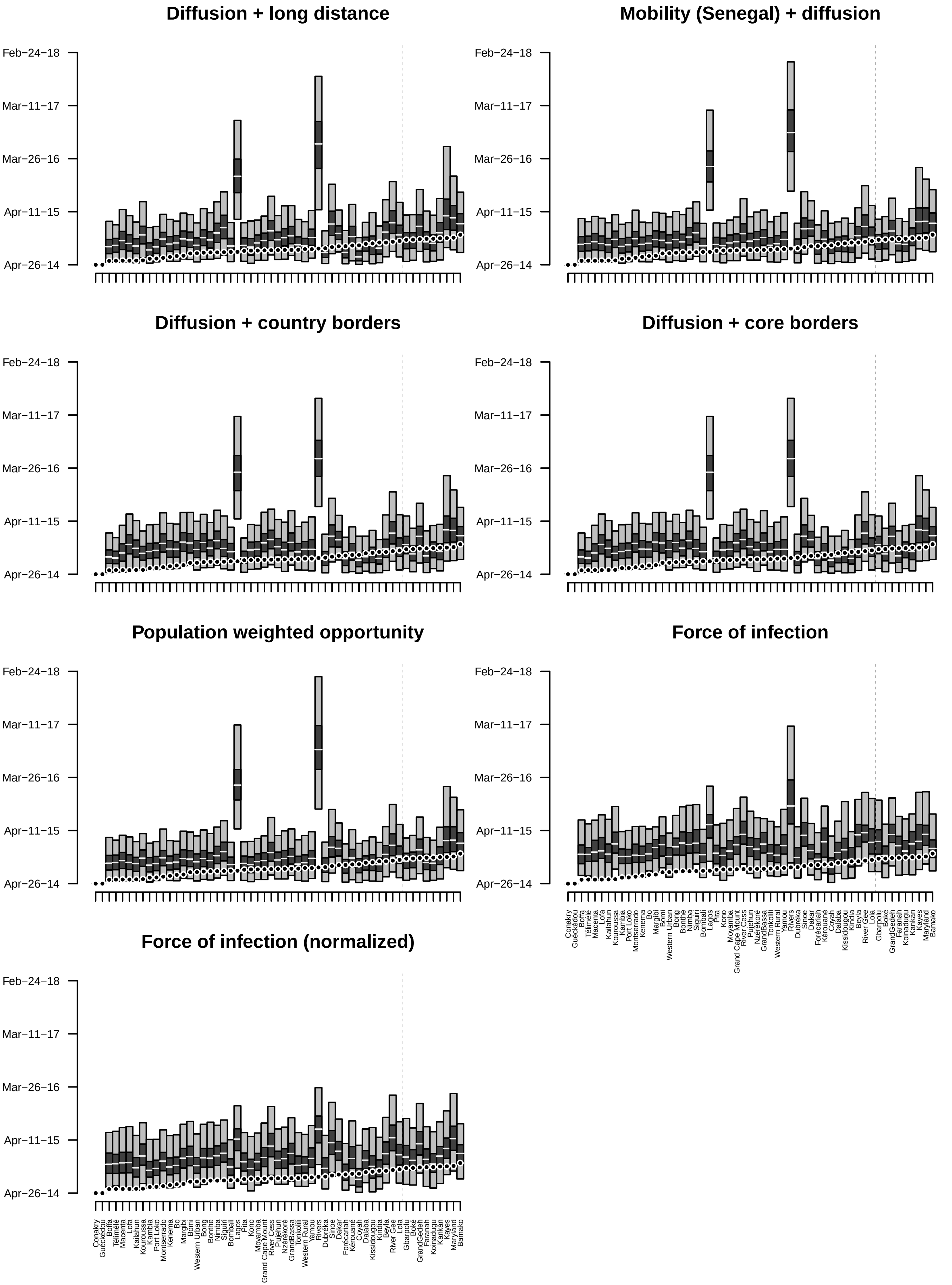

Diffusion + long distance
Mobility (Senegal) + diffusion
Diffusion + country borders
Diffusion + core borders
Population weighted opportunity
Force of infection
Force of infection (normalized)
Feb−24−18
Mar−11−17
Mar−26−16
Apr−11−15
Apr−26−14
Conakry
Guéckédou
Boffa
Télimélé
Macenta
Lofa
Kailahun
Kouroussa
Kambia
Port Loko
Montserrado
Kenema
Bo
Margibi
Bomi
Western Urban
Bong
Bonthe
Nimba
Siguiri
Bombali
Lagos
Pita
Kono
Moyamba
Grand Cape Mount
River Cess
Pujehun
Nzérékoré
GrandBassa
Tonkolili
Western Rural
Yamou
Rivers
Dubréka
Sinoe
Dakar
Forécariah
Kérouané
Coyah
Dalaba
Kissidougou
Kindia
Beyla
River Gee
Lola
Gbarpolu
Boké
GrandGedeh
Faranah
Koinadugu
Kankan
Kayes
Maryland
Bamako

Figure S3:
Coverage vs. correlation of unit-infection dates. The correlation between the actual infection date and simulated median date of infection for a representative subset of compared models is plotted versus the coverage for the same model. Coverage is defined as percent of actual infection dates occurring in the 95% prediction intervals (Fig S2). The best-fit and other gravity models have the best combination of accuracy and precision by these measures.

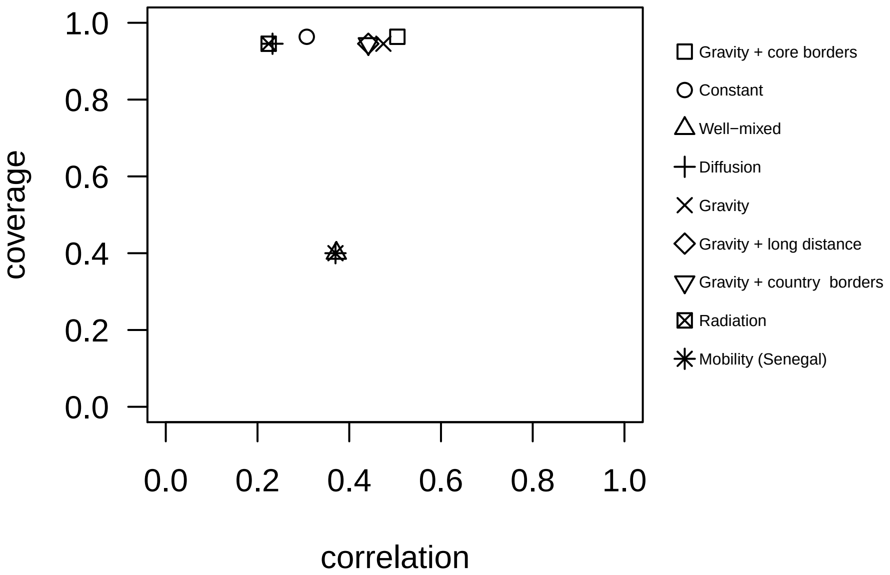
coverage
1.0
0.8
0.6
0.4
0.2
0.0
0.0
0.2
0.4
0.6
0.8
1.0
correlation
Gravity + core borders
Constant
Well−mixed
Diffusion
Gravity
Gravity + long distance
Gravity + country borders
Radiation
Mobility (Senegal)

Figure S4:
Macro-scale spread statistics of gravity + core border model. Orange shading represents simulated spread from April 24th to Oct 1st, 2014 and unshaded represents forecasts obtained by forward simulations until June 30th 2015. Simulations forward were based on epidemic transmission remaining unchanged from the fitting period and therefore does not account for the realized success of control. Dots are observed data, solid lines are median values, and dotted lines/shaded areas represent 95% prediction intervals. a) The number of newly infected administrative units is consistent with observation and continues to accelerate if spread continues uncontrolled. The maximum b) and median c) distance from Guéckédou; the maximum distance increased faster than predicted due to early spread to Nigeria. d) The convex hull of infection area is the total area within the minimum convex polygon of infected unit centroids.

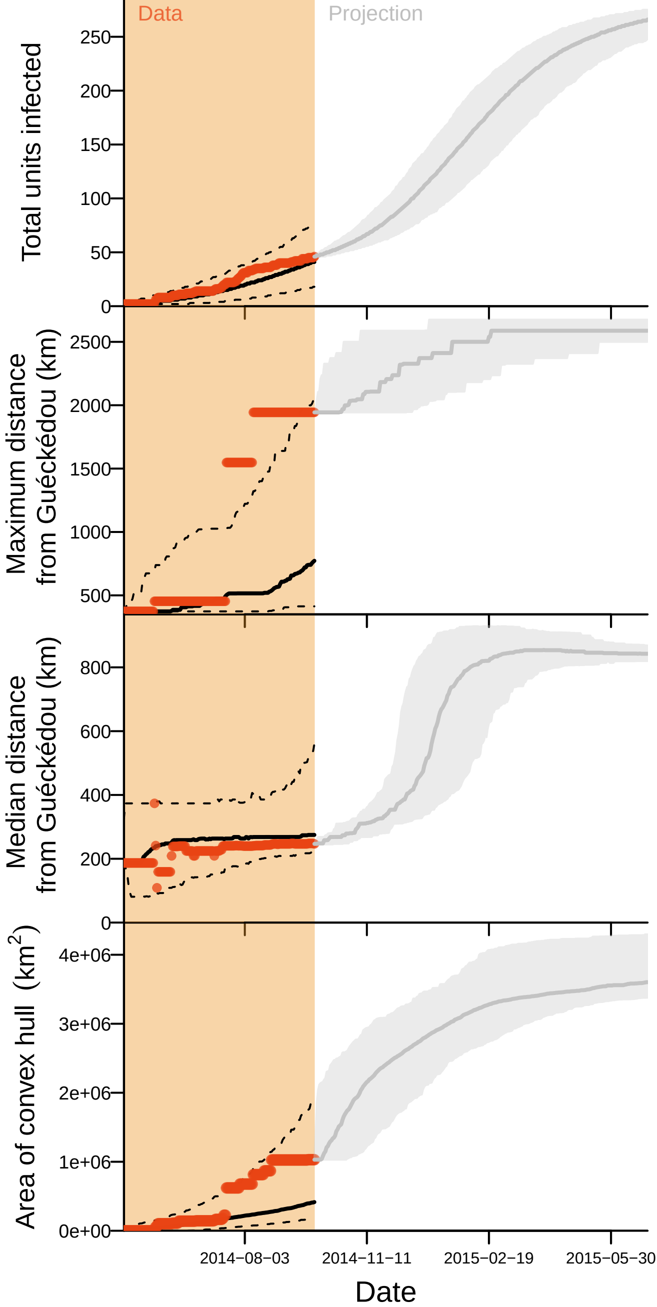

Gravity + core borders

Figure S5:

Predicted day of infection for infected administrative units based on the best fit model. Predicted intervals for the counties without observed infections are included for assessment of lack of spread. The predicted day of infection for nodes is based on simulations from April 24th. For each node, the 95% prediction interval is in light blue with the interquartile range specified as dark blue and the median infection as a break. Dots represent the observed day of infection for each node colored by country. Infection locations used in fitting are to the left of the dotted line and infection subsequent to Oct 1 are to the right of the first dotted line. After the second dotted line are the counties in the dataset that were not infected. Most have median infection dates much later than predicted for the infected counties and the actual infections also fall below the interquartile range of these counties. Given that interventions and changes in behavior began to slow the outbreak in late 2015 it is unreasonable to expect many additional infections after this point. However, it is interesting that much of the remainder of uninfected West African locations have similar, wide ranges of the predicted date of infection, this illustrates the importance of stochasticity in determining the realized path of spread. We have further illustrated the difference between core-countries and the rest of the region by coloring core-countries with a lighter color.

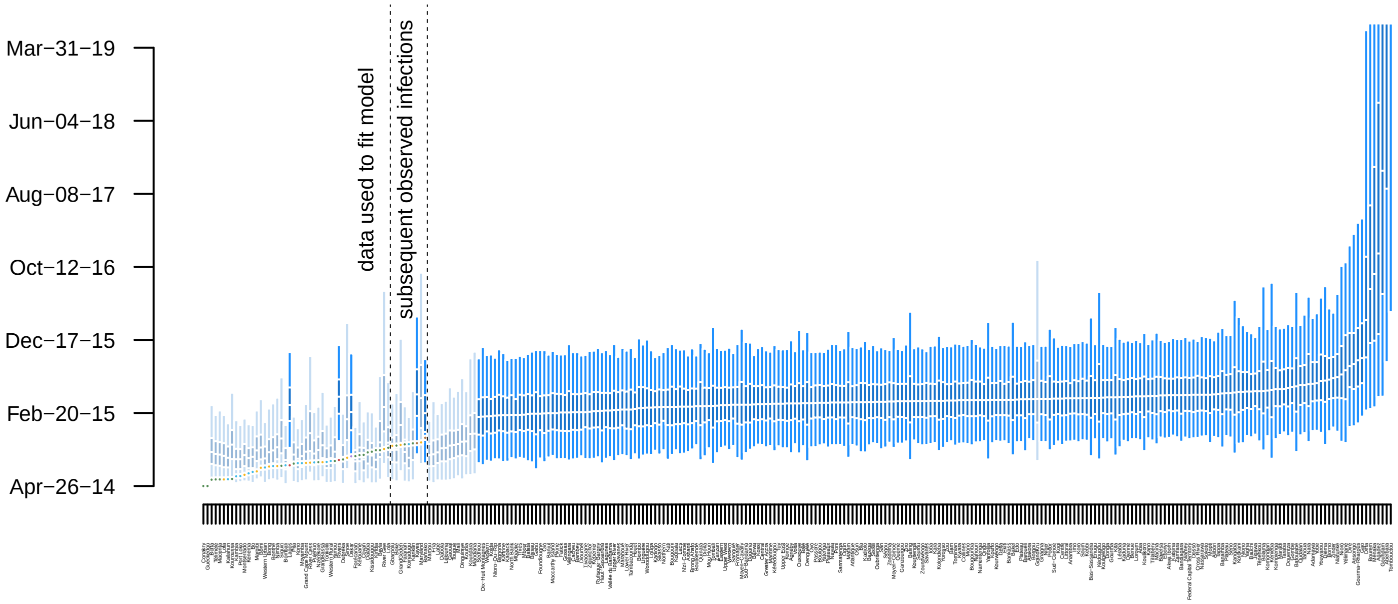

Mar−31−19
Jun−04−18
Aug−08−17
Oct−12−16
Dec−17−15
Feb−20−15
Apr−26−14
data used to fit model
subsequent observed infections

Supplementary Information Table 1: Infection data and sources.

The following details the sources of information used to determine the initial dates of infection for each administrative unit used in model fitting. Information was accumulated and updated as the epidemic progressed and dates used for fitting were finalized Oct 10th, 2014. Recently WHO has released new data showing confirmed infections in counties earlier (and in a few cases later) than the data below. These new data also differed in being aggregated weekly. We used these recent patient databases to fit the same models to weekly data and found qualitatively similar results with the same best fit model and the same support for gravity models overall with AIC.

Country	County	Date	Official Confirmation	Additional Source
Sierra Leone	Kailahun	5/24/2014	WHO Outbreak News[1], WHO Essay[2]	
Sierra Leone	Kenema	6/17/2014	24 June GoSL SitRep[3]	Media[4]
Sierra Leone	Kono	7/29/2014	NGO (Wellbody Alliance)[5]	
Sierra Leone	Bombali	7/20/2014	30 July IFRC-SL SitRep[6]	Media[7,8]
Sierra Leone	Kambia	6/5/2014	6 June GoSL SitRep[9]	
Sierra Leone	Port Loko	6/6/2014	11 June GoSL SitRep[10]	Media[11]
Sierra Leone	Tonkolili	8/3/2014	4 August GoSL SitRep[12]	
Sierra Leone	Bo	6/23/2014	24 June GoSL SitRep[3]	
Sierra Leone	Bonthe	7/17/2014	20 July US Embassy[13]	Blog (text of SL Facebook post)[14]
Sierra Leone	Moyamba	7/29/2014	4 August GoSL SitRep[12]	Media[15]
Sierra Leone	Pujehun	8/1/2014	4 August GoSL SitRep[12]	Media[16]
Sierra Leone	Western Rural	8/7/2014	8 August GoSL SitRep[17]	
Sierra Leone	Western Urban	7/11/2014	20 July US Embassy[13]	Media[18]
Guinea	Boffa	5/22/2014	6 June GoG SitRep[19]	
Guinea	Conakry	4/26/2014		
Guinea	Kissidougou	9/14/2014	16 September GoG SitRep[20]	
Guinea	Krouan	8/31/2014	31 August GoG SitRep[21]	
Guinea	Kouroussa	5/25/2014	5 June GoG SitRep[19]	
Guinea	Siguiri	7/18/2014	22 July GoG SitRep[22]	
Guinea	Coyah	9/1/2014	2 September GoG SitRep[23]	
Guinea	Dubrka	8/13/2014	14 August GoG SitRep[21]	
Guinea	Forcariah	8/27/2014	29 August GoG SitRep[24]	
Guinea	Kindia	9/20/2014	20 September GoG SitRep[25]	
Guinea	Tliml	5/23/2014	WHO Outbreak News[26], 27 May GoG SitRep[27]	
Guinea	Dalaba	9/11/2014	12 September GoG SitRep[28]	
Guinea	Pita	7/27/2014	4 August GoG SitRep[29]	Media[30]
Guinea	Beyla	9/20/2014	25 September GoG SitRep[31]	
Guinea	Guckdou	4/26/2014		
Guinea	Lola	9/30/2014	1 October GoG SitRep[32]	
Guinea	Macenta	5/23/2014	WHO Outbreak News[26], 27 May GoG SitRep[27]	
Guinea	Nzrkor	8/2/2014	4 August GoG SitRep[29]	
Guinea	Yamou	8/7/2014	7 August GoG SitRep[33]	
Liberia	Nimba	7/17/2014	20 July GoL SitRep[34], 16 July UNICEF SitRep[35]	
Liberia	River Cess	7/31/2014	31 July GoL SitRep[36], 15 August UNICEF SitRep[37]	
Liberia	River Gee	9/25/2014	25 September GoL SitRep[38]	
Liberia	Sinoe	8/20/2014	20 August GoL SitRep[39]	
Liberia	Bomi	7/10/2014	10 July GoL SitRep	

Liberia	Bong	7/15/2014	16 July UNICEF SitRep[35]	
Liberia	Grand Cape Mount	7/30/2014	31 July GoL SitRep[36], 2 August GoL SitRep[40]	
Liberia	Grand Bassa	8/2/2014	2 August GoL SitRep[40] Probable, WHO Patient-level: there are earlier cases[41], GoL Ministry of Information[42]	Media[43,44]
Liberia	Lofa	5/23/2014	6 June GoL SitRep[45]	
Liberia	Margibi	6/25/2014	25 June GoL SitRep[46]	
Liberia	Montserrado	6/11/2014	11 June GoL SitRep[47], 12 June GoL SitRep[48]	
Nigeria	Lagos	7/20/2014	WHO Outbreak News[49]	
Nigeria	Rivers	8/11/2014	WHO Situation Assessment[50]	
Senegal	Dakar	8/26/2014	WHO Outbreak News[51]	

1. Ebola virus disease, West Africa (Update of 26 May 2014) - WHO | Regional Office for Africa. *WHO | Regional Office for Africa* (2014). at <http://www.afro.who.int/en/clusters-a-programmes/dpc/epidemic-a-pandemic-alert-and-response/outbreak-news/4143-ebola-virus-disease-west-africa-26-may-2014.html>
2. WHO | Sierra Leone: a traditional healer and a funeral. *WHO* (2014). at <http://who.int/csr/disease/ebola/ebola-6-months/sierra-leone/en/>
3. *EBOLA VIRUS DISEASE Sierra Leone Situation Report (SitRep) – 24 June 2014*. (Government of Sierra Leone Ministry of Health and Sanitation, 2014). at <http://www.humanitarianresponse.info/operations/sierra-leone/document/ebola-virus-disease-sierra-leone-situation-report-sitrep-%E2%80%93-24-june>
4. Moriba, S. B. Sierra Leone News: Kenema Matron calls for support to tackle Ebola « Awoko Newspaper. (2014). at <http://awoko.org/2014/06/19/sierra-leone-news-kenema-matron-calls-for-support-to-tackle-ebola/>
5. First confirmed case of Ebola in Kono. *Wellbody Alliance* (2014). at <http://wellbodyalliance.org/blog/2014/jul/29/first-confirmed-case-ebola-kono/>
6. Emergency appeal operations update Sierra Leone: Ebola Virus Disease. *Appeals - IFRC* (2014). at <http://www.ifrc.org/en/publications-and-reports/appeals/?ac=&at=0&c=213&co=SP161SL&dt=1&f=&p=0&re=&sort=Date&t=&ti=&zo=>
7. Augustine Samba. As only Kono, Tonkolili, Koinadugu & Moyamba are Free, Bombali Confirms Ebola cases in Makari – Gbanti. *Awareness Times: Sierra Leone News & Information* (2014). at <http://news.sl/drwebsite/publish/article_200525859.shtml>
8. Mohamed Kabba. Bombali Ebola: Five Dead, 6 more Suspects Confirmed. *Awoko Newspaper* (2014). at <http://awoko.org/2014/07/31/sierra-leone-news-bombali-ebola-five-dead-6-more-suspects-confirmed/>
9. *EBOLA VIRUS DISEASE Sierra Leone Situation Report (SitRep) – 6 June 2014*. (Government of Sierra Leone Ministry of Health and Sanitation, 2014).
10. *EBOLA VIRUS DISEASE Sierra Leone Situation Report (SitRep) – 11 June 2014*. (Government of Sierra Leone Ministry of Health and Sanitation, 2014).
11. Hassan Bruz. EBOLA Travels from the east to the north- Where next? *My Sierra Leone* (2014). at <http://mysierraleoneonline.com/sl_portal/site/news/detail/2549>

12. *EBOLA VIRUS DISEASE Sierra Leone Situation Report (SitRep) – 04 August 2014*. (Government of Sierra Leone Ministry of Health and Sanitation, 2014). at <http://www.humanitarianresponse.info/system/files/documents/files/Sierra%20Leone%20Ebola%20sitrep%2004%20August%202014.pdf>
13. As of July 20, there are 405 confirmed cases of Ebola Virus Disease in Sierra Leone. *Facebook| U.S. Department of State: Embassy Freetown, Sierra Leone* (2014). at <https://www.facebook.com/sierraleone.usembassy/posts/10152543703407866>
14. H5N1: Sierra Leone: Ebola update, July 17. (2014). at <http://crofsblogs.typepad.com/h5n1/2014/07/sierra-leone-ebola-update-july-17.html>
15. Koroma, N. A. Ebola Kills Four in Bonthe, Moyamba. *Premier Media Sierra Leone* (2014). at <http://www.premiermedia.sl/content/ebola-kills-four-bonthe-moyamba>
16. Abibatu Kamara. Ebola reaches Pujehun. *Awoko Newspaper* (2014). at <http://awoko.org/2014/08/05/sierra-leone-news-ebola-reaches-pujehun/>
17. *EBOLA VIRUS DISEASE Sierra Leone Situation Report (SitRep) – 08 August 2014*. (Government of Sierra Leone Ministry of Health and Sanitation, 2014). at <http://www.humanitarianresponse.info/system/files/documents/files/Sierra%20Leone%20Ebola%20sitrep%2008%20August%202014.pdfhttp://www.humanitarianresponse.info/system/files/documents/files/Sierra%20Leone%20Ebola%20sitrep%2008%20August%202014.pdf>
18. Silas Gbandia. Ebola Spreads to Sierra Leone Capital of Freetown as Deaths Rise. *Bloomberg News* (2014). at <http://www.bloomberg.com/news/2014-07-12/ebola-spreads-to-sierra-leone-capital-of-freetown-as-deaths-rise.html>
19. *Rapport de la Situation Epidémiologique Guinée, Epidémie de la Fièvre Hémorragique Ebola Situation au 05 juin 2014*. (Government of Guinea, 2014). at <http://www.humanitarianresponse.info/system/files/documents/files/Guinea_Ebola_Sit_Rep_n%C2%B0%2055%20du%2005_juin_2014.pdf.pdf>
20. *Rapport de la Situation Epidémiologique Guinée, Epidémie de la Fièvre Hémorragique Ebola Situation au 16 Septembre 2014 à 18h00*. (Government of Guinea, 2014). at

<http://www.humanitarianresponse.info/system/files/documents/files/GUINEA_EBOLA_SITREP%20N%20154%20DU%2017%20SEPTEMBRE_2014.docx%20VF.pdf>

21. *Rapport de la Situation Epidémiologique Guinée, Epidémie de la Fièvre Hémorragique Ebola Situation au 31 août 2014 à 18h00*. (Government of Guinea, 2014). at <http://www.humanitarianresponse.info/system/files/documents/files/GUINEA_EBOLA_SITREP%20N%20138%20DU%2001%20SEPTEMBRE_2014%20V3.pdf>
22. *Rapport de la Situation Epidémiologique Guinée, Epidémie de la Fièvre Hémorragique Ebola Situation au 22 juillet 2014 à 18h00*. (Government of Guinea, 2014). at <http://www.humanitarianresponse.info/system/files/documents/files/Guinea_Ebola_Sit_Rep_n98%20du%2023%20juillet_2014.pdf>
23. *Rapport de la Situation Epidémiologique Guinée, Epidémie de la Fièvre Hémorragique Ebola Situation au 02 Septembre 2014 à 18h00*. (Government of Guinea, 2014). at <http://www.humanitarianresponse.info/system/files/documents/files/GUINEA_EBOLA_SITREP%20N%20139%20DU%2002%20SEPTEMBRE_2014.pdf>
24. *Rapport de la Situation Epidémiologique Guinée, Epidémie de la Fièvre Hémorragique Ebola Situation au 29 août 2014 à 18h00*. (Government of Guinea, 2014). at <http://www.humanitarianresponse.info/system/files/documents/files/GUINEA_EBOLA_SITREP%20N%20136%20DU%2030%20AOUT_2014.VF_.pdf>
25. *Rapport de la Situation Epidémiologique Guinée, Epidémie de la Fièvre Hémorragique Ebola Situation au 20 Septembre 2014 à 18h00*. (Government of Guinea, 2014). at <http://www.humanitarianresponse.info/system/files/documents/files/GUINEA_EBOLA_SITREP%20N%20158%20DU%2021%20SEPTEMBRE_2014.docx%20VF.pdf>
26. Ebola virus disease, West Africa (Situation as of 23 May 2014). *WHO | Regional Office for Africa* (2014). at <http://www.afro.who.int/en/clusters-a-programmes/dpc/epidemic-a-pandemic-alert-and-response/outbreak-news/4141-ebola-virus-disease-west-africa-situation-as-of-23-may-2014.html>
27. *Rapport de la Situation Epidémiologique, Guinée, Epidémie de la Fièvre Hémorragique Ebola, Situation au 27 mai 2014.* (Government of Guinea, 2014). at

<http://www.humanitarianresponse.info/system/files/documents/files/Guinea_Ebola_Sit_Rep_n%2048%20du%2027_mai_2014.pdf>

28. *Rapport de la Situation Epidémiologique Guinée, Epidémie de la Fièvre Hémorragique Ebola Situation au 12 Septembre 2014 à 18h00*. (Government of Guinea, 2014). at <http://www.humanitarianresponse.info/system/files/documents/files/GUINEA_EBOLA_SITREP%20N%20150%20DU%2013%20SEPTEMBRE_2014.VF_.pdf>
29. *Rapport de la Situation Epidémiologique Guinée, Epidémie de la Fièvre Hémorragique Ebola Situation au 04 août 2014 à 18h00*. (Government of Guinea, 2014). at <http://www.humanitarianresponse.info/system/files/documents/files/GUINEA_EBOLA_SITREP%20N%20111%20DU%2005%20AOUT_2014%20VFINALE.PDF>
30. Bah, A. Une victime d'Ebola en provenance de la Sierra Leone inhumée à Pita. *Guinéenews* (2014). at <http://guineenews.org/une-victime-debola-en-provenance-de-la-sierra-leone-inhumee-a-pita/>
31. *Rapport de la Situation Epidémiologique Guinée, Epidémie de la Fièvre Hémorragique Ebola Situation au 25 Septembre 2014 à 18h00*. (Government of Guinea, 2014). at <http://www.humanitarianresponse.info/system/files/documents/files/GUINEA_EBOLA_SITREP%20N%20163%20DU%2026%20SEPTEMBRE_2014%20VF.pdf>
32. *Rapport de la Situation Epidémiologique Guinée, Epidémie de la Fièvre Hémorragique Ebola Situation au 01 Octobre 2014 à 18h00*. (Government of Guinea, 2014). at <http://www.humanitarianresponse.info/system/files/documents/files/GUINEA_EBOLA_SITREP%20N%20169%20DU%2001%20OCTOBRE_2014%20VF.pdf>
33. *Rapport de la Situation Epidémiologique Guinée, Epidémie de la Fièvre Hémorragique Ebola Situation au 07 août 2014 à 18h00*. (Government of Guinea, 2014). at <http://www.humanitarianresponse.info/system/files/documents/files/GUINEA_EBOLA_SITREP%20N%20114%20DU%2008%20AOUT_2014%20VF.PDF>
34. *Liberia Ebola SitRep no. 67*. (Ministry of Health and Social Welfare, Republic of Liberia, 2014). at <http://www.mohsw.gov.lr/documents/Liberia%20Ebola%20SitRep%2067%20%20July%2020%202014.pdf>
35. *UNICEF-Liberia Ebola Virus Disease: SitRep #37, 16 July 2014*. (United Nations Children's Fund, 2014). at <http://www.unicef.org/appeals/files/UNICEF_Liberia_SitRep_Ebola_Viral_Disease_16_July_2014.pdf>

36. *Liberia Ebola SitRep no. 77*. (Ministry of Health and Social Welfare, Republic of Liberia, 2014). at <http://www.mohsw.gov.lr/documents/Liberia%20Ebola%20SitRep%2077%20July%2031%202014.pdf>
37. *UNICEF-Liberia Ebola Virus Disease: SitRep #47 15 August 2014*. (United Nations Children's Fund, 2014). at <http://www.unicef.org/appeals/files/UNICEF_Liberia_Ebola_Viral_Disease_SitRep47_15_August_2014.pdf>
38. *Liberia Ebola SitRep no. 133*. (Ministry of Health and Social Welfare, Republic of Liberia, 2014). at <http://www.mohsw.gov.lr/documents/Liberia%20Ebola%20Sit%20Rep%20133%20Sept%2025.%202014%20%282%29.pdf>
39. *Liberia Ebola SitRep no. 97*. (Ministry of Health and Social Welfare, Republic of Liberia, 2014). at <http://www.mohsw.gov.lr/documents/Liberia%20Ebola%20SitRep%2097%20Aug%2020,%202014.pdf>
40. *Liberia Ebola SitRep no. 79*. (Ministry of Health and Social Welfare, Republic of Liberia, 2014). at <http://mohsw.gov.lr/documents/Liberia%20Ebola%20SitRep%2079%20Aug%202,%202014.pdf>
41. Ebola data and statistics. at <http://apps.who.int/gho/data/view.ebola-sitrep.ebola-country-LBR-new-conf-prob-districs-20150107-data?lang=en>
42. Government of the Republic of Liberia (Ministry of Information) - Bassa Task Force Notes Six Confirmed Ebola Deaths. *Government of Liberia: Ministry of Information* (2014). at <http://www.micatliberia.com/index.php/ebola-update/1146-bassa-task-force-notes-six-confirmed-ebola-deaths.html>
43. Liberia: First Bassa Ebola Case Confirmed. *The New Republic Liberia* (8/5//14). at <http://allafrica.com/stories/201408051284.html>
44. FrontPageAfrica - Ebola Claims One Life In Grand Bassa, Five Suspected Cases. *Front Page Africa* (2014). at <http://frontpageafricaonline.com/index.php/county-news/2525-ebola-claims-one-life-in-grand-bassa-five-suspected-cases>
45. *EBOLA VIRUS DISEASE EPIDEMIC IN LIBERIA Situation Report (Sitrep No.26) as at 09:00 Hrs; 6th June, 2014*. (Ministry of Health and Social Welfare, Republic of Liberia, 2014). at <http://www.mohsw.gov.lr/documents/Liberia%20Ebola%20Virus%20Disease%20Situation%20Report-No-%2026%20as%20of%20the%206-06-14.pdf>

46. *RE-OCCURANCE OF EBOLA VIRUS DISEASE EPIDEMIC IN LIBERIA SINCE THE 29th MAY, 2014: Edition: No. 42. Reporting period: June 25th 2014 as at 21:00 Hours*. (Ministry of Health and Social Welfare, Republic of Liberia, 2014). at <http://www.mohsw.gov.lr/content_display.php?press_id=69&sub=press_release>
47. *SITUATION REPORT EBOLA VIRUS DISEASE EPIDEMIC IN LIBERIA Edition: No. 30. Reporting period: as at 21:00 Hrs.; 11th June 2014*. (Ministry of Health and Social Welfare, Republic of Liberia, 2014). at <http://mohsw.gov.lr/documents/Liberia%20Ebola%20Viral%20Disease-Sit%20Rep-%2030-%2011th%20June%202014.pdf%20docx%20(1).pdf>
48. *SITUATION REPORT EBOLA VIRUS DISEASE EPIDEMIC IN LIBERIA Edition: No. 31. Reporting time: as at 21:00 Hrs.; 12th June 2014*. (Ministry of Health and Social Welfare, Republic of Liberia, 2014). at <http://www.mohsw.gov.lr/documents/12juneLiberia%20Ebola%20Viral%20Disease-Sit%20Rep-%2031-%2012th%20June%202014%20pdf%20docx%20(1).pdf>
49. Ebola virus disease, West Africa – update 29 July 2014 - WHO | Regional Office for Africa. *WHO | Regional Office for Africa* (2014). at <http://www.afro.who.int/en/clusters-a-programmes/dpc/epidemic-a-pandemic-alert-and-response/outbreak-news/4236-ebola-virus-disease-west-africa-29-july-2014.html>
50. WHO | Ebola situation in Port Harcourt, Nigeria. *WHO* (2014). at <http://www.who.int/mediacentre/news/ebola/3-september-2014/en/>
51. WHO | Ebola virus disease update - Senegal. (2014). at <http://www.who.int/csr/don/2014_08_30_ebola/en/>

Table S2: Model comparison

Model name	Infection probability	NLL	AIC
Constant	β_0 (no network)	346.1	348
Well-mixed	β_0	350.7	353
Diffusion	$\frac{1}{1+e^{\beta_0+\beta_1 d_{ij}}}$	281.9	353
Diffusion + long distance	$\frac{1}{1+e^{\beta_0+\beta_1 {d_{ij}}^{\beta_2}}}$	275.1	281
Gravity	$\frac{1}{1+e^{\beta_0+\beta_1 \frac{d_{ij}}{(p_i p_j)^{B_2}}}}$	273.2	279
Gravity + long distance	$\frac{1}{1+e^{\beta_0+\beta_1 \frac{{d_{ij}}^{\beta_2}}{(p_i p_j)^{B_3}}}}$	272.2	280
Mobility (Senegal)	$\frac{1}{1+e^{\beta_0+\beta_1 \text{mob}}}$	350.4	354
Mobility (Senegal) + diffusion	$\frac{1}{1+e^{\beta_0+\beta_1 \text{mob}+\beta_2 d_{ij}}}$	281.8	288
Diffusion + country borders	$\begin{cases} \beta_2 \frac{1}{1+e^{\beta_0+\beta_1 d_{ij}}} & \text{if crossing border} \\ \frac{1}{1+e^{\beta_0+\beta_1 d_{ij}}} & \text{if not crossing border} \end{cases}$	280.0	286
Diffusion + core borders	$\begin{cases} \beta_2 \frac{1}{1+e^{\beta_0+\beta_1 d_{ij}}} & \text{if crossing core border} \\ \frac{1}{1+e^{\beta_0+\beta_1 d_{ij}}} & \text{if not crossing core border} \end{cases}$	274.9	281
Gravity + country borders	$\begin{cases} \beta_3 \frac{1}{1+e^{\beta_0+\beta_1 \frac{d_{ij}}{(p_i p_j)^{B_2}}}} & \text{if crossing border} \\ \frac{1}{1+e^{\beta_0+\beta_1 \frac{d_{ij}}{(p_i p_j)^{B_2}}}} & \text{if not crossing border} \end{cases}$	272.5	281
Gravity + core borders	$\begin{cases} \beta_3 \frac{1}{1+e^{\beta_0+\beta_1 \frac{d_{ij}}{(p_i p_j)^{B_2}}}} & \text{if crossing core border} \\ \frac{1}{1+e^{\beta_0+\beta_1 \frac{d_{ij}}{(p_i p_j)^{B_2}}}} & \text{if not crossing core border} \end{cases}$	270.0	278
Radiation	$\frac{1}{1+e^{\beta_0+\beta_1 d_{ij}+\beta_2 \text{rad}}}$	281.8	288

Population weighted opportunity	$\dfrac{1}{1+e^{\beta_0+\beta_1 d_{ij}+\beta_2 \text{ pwo}}}$	281.9	288
Force of infection (unnormalized)	$1-e^{\left(\beta_0 p_i^{\beta_1} \sum_j \frac{p_j^{B_2}}{d_{ij}^{\beta_3}}\right)}$ (no network)	275.4	283
Force of infection (normalized)	$1-e^{\left(\frac{\beta_0 p_i^{\beta_1} \sum_j \frac{p_j^{B_2}}{d_{ij}^{\beta_3}}}{\left(\sum_{k\neq i} \frac{p_k^{B_2}}{d_{ik}^{\beta_3}}\right)^{\beta_4}}\right)}$ (no network)	274.0	284

Table S3: Estimated parameter values

Model name	Infection probability	β_0	β_1	β_2	β_3	β_4
Constant	β_0 (no network)	0.0010				
Well-mixed	β_0	0.00005				
Diffusion	$\frac{1}{1+e^{\beta_0+\beta_1 d_{ij}}}$	5.630	1.032			
Diffusion + long distance	$\frac{1}{1+e^{\beta_0+\beta_1 {d_{ij}}^{\beta_2}}}$	0.876	5.748	0.341		
Gravity	$\frac{1}{1+e^{\beta_0+\beta_1 \frac{d_{ij}}{(p_i p_j)^{B_2}}}}$	5.246	155.8	0.186		
Gravity + long distance	$\frac{1}{1+e^{\beta_0+\beta_1 \frac{{d_{ij}}^{\beta_2}}{(p_i p_j)^{B_3}}}}$	3.659	22.63	0.593	0.080	
Mobility (Senegal)	$\frac{1}{1+e^{\beta_0+\beta_1 \text{mob}}}$	9.886	0.000007			
Mobility (Senegal) + diffusion	$\frac{1}{1+e^{\beta_0+\beta_1 \text{mob}+\beta_2 d_{ij}}}$	5.594	0.000003	1.046		
Diffusion + country borders	$\begin{cases} \beta_2 \frac{1}{1+e^{\beta_0+\beta_1 d_{ij}}} & \text{if crossing border} \\ \frac{1}{1+e^{\beta_0+\beta_1 d_{ij}}} & \text{if not crossing border} \end{cases}$	5.561	0.806	0.257		
Diffusion + core borders	$\begin{cases} \beta_2 \frac{1}{1+e^{\beta_0+\beta_1 d_{ij}}} & \text{if crossing core border} \\ \frac{1}{1+e^{\beta_0+\beta_1 d_{ij}}} & \text{if not crossing core border} \end{cases}$	6.484	0.510	0.070		

Model name	Infection probability	β_0	β_1	β_2	β_3	β_4
Gravity + country borders	$\begin{cases} \beta_3 \dfrac{1}{1+e^{\beta_0+\beta_1 \frac{d_{ij}}{(p_i p_j)^{B_2}}}} & \text{if crossing border} \\ \dfrac{1}{1+e^{\beta_0+\beta_1 \frac{d_{ij}}{(p_i p_j)^{B_2}}}} & \text{if not crossing border} \end{cases}$	5.166	157.1	0.189	0.507	
Gravity + core borders	$\begin{cases} \beta_3 \dfrac{1}{1+e^{\beta_0+\beta_1 \frac{d_{ij}}{(p_i p_j)^{B_2}}}} & \text{if crossing core border} \\ \dfrac{1}{1+e^{\beta_0+\beta_1 \frac{d_{ij}}{(p_i p_j)^{B_2}}}} & \text{if not crossing core border} \end{cases}$	5.792	105.7	0.186	0.150	
Radiation	$\dfrac{1}{1+e^{\beta_0+\beta_1 d_{ij}+\beta_2 \,\text{rad}}}$	5.588	1.046	0.000002		
Population weighted opportunity	$\dfrac{1}{1+e^{\beta_0+\beta_1 d_{ij}+\beta_2 \,\text{pwo}}}$	5.606	1.041	0.0000006		
Force of infection (unnormalized)	$1-e^{\left(\beta_0 p_i^{\beta_1} \sum_j \frac{p_j^{B_2}}{d_{ij}^{\beta_3}}\right)}$ (no network)	-12.14	0.544	-1.267	2.399	
Force of infection (normalized)	$1-e^{\left(\frac{\beta_0 p_i^{\beta_1} \sum_j \frac{p_j^{B_2}}{d_{ij}^{\beta_3}}}{\left(\sum_{k\neq i} \frac{p_k^{B_2}}{d_{ik}^{\beta_3}}\right)^{\beta_4}}\right)}$ (no network)	-0.113	0.395	-1.075	2.621	0.404

Table S4: Model parameter confidence ranges

Model name	Infection probability	Δ_0	Δ_1	Δ_2
Constant	β_0 (no network)	0.0000002		
Well-mixed	β_0	0.0000000004		
Diffusion	$\frac{1}{1+e^{\beta_0+\beta_1 d_{ij}}}$	1.079	0.502	
Diffusion + long distance	$\frac{1}{1+e^{\beta_0+\beta_1 d_{ij}^{\beta_2}}}$	8.841	9.049	0.450
Diffusion + country borders	$\begin{cases} \beta_2 \frac{1}{1+e^{\beta_0+\beta_1 d_{ij}}} & \text{if crossing border} \\ \frac{1}{1+e^{\beta_0+\beta_1 d_{ij}}} & \text{if not crossing border} \end{cases}$	0.669	0.411	0.397
Diffusion + core borders	$\begin{cases} \beta_2 \frac{1}{1+e^{\beta_0+\beta_1 d_{ij}}} & \text{if crossing core border} \\ \frac{1}{1+e^{\beta_0+\beta_1 d_{ij}}} & \text{if not crossing core border} \end{cases}$	6.484	0.432	0.146

Δ values represent the confidence interval on either side of the estimated β values (see Table S2).
The remainder of the confidence intervals could not be estimated with this approach (see Methods).

Table S5A: Spearman correlation of selected transmission links

	Gravity + core borders	Gravity + long distance	Gravity	Gravity + country borders	Diffusion	Radiation
Gravity + long distance	0.968					
Gravity	0.981	0.991				
Gravity + country borders	0.982	0.990	0.999			
Diffusion	0.852	0.938	0.886	0.883		
Radiation	0.852	0.938	0.886	0.883	0.999	
Mobility (Senegal)	0.974	0.954	0.984	0.985	0.797	0.797

Table S5B: Pearson correlation of selected transmission links

	Gravity + core borders	Gravity + long distancel	Gravity	Gravity + country borders	Diffusion	Radiation
Gravity + long distancel	0.959					
Gravity	0.983	0.972				
Gravity + country borders	0.983	0.973	0.999			
Diffusion	0.809	0.908	0.834	0.830		
Radiation	0.809	0.908	0.834	0.830	0.999	
Mobility (Senegal)	0.151	0.188	0.146	0.150	0.135	0.133
